# Toxicogenetics: in search of host susceptibility to environmental toxicants

**DOI:** 10.3389/fgene.2014.00327

**Published:** 2014-09-22

**Authors:** Gelareh Alam, Byron C. Jones

**Affiliations:** Department of Biobehavioral Health, The Pennsylvania State University, University Park, PAUSA

**Keywords:** systems genetics, forward genetic analysis, reverse genetic analysis

## Abstract

Heavy metals, various pesticide and herbicides are implicated as risk factors for human health. Paraquat, maneb, and rotenone, carbamate, and organophosphorous insecticides are examples of toxicants for which acute and chronic exposure are associated with multiple neurological disorders including Parkinson’s disease. Nevertheless, the role of pesticide exposure in neurodegenerative diseases is not clear-cut, as there are inconsistencies in both the epidemiological and preclinical research. The aim of this short review is to show that at least, some of the inconsistencies are related to individual differences in susceptibility to the effects of neurotoxicants, individual differences that can be traced to the genetic constitution of the individuals and animals studies, i.e., host-based susceptibility.

## INTRODUCTION

### EPIDEMIOLOGICAL STUDIES

At least two major neurodegenerative diseases, Alzheimer’s and Parkinson’s, can be classified as early or late onset. Early onset disease (i.e., prior to age 50) is seen as less frequent than late onset and can often be tied to specific genetic factors (e.g., Jones et al., 2014a). The etiology of later onset disease is less clear and very likely a result of genes interacting with the environment. Parkinson’s disease (PD) is characterized by a loss of dopamine (DA) neurons in the substantia nigra pars compacta and subsequent loss of DA function in the projection area, namely the striatum.

A number of researchers have reported the association between exposure insecticides and herbicides as increasing risk for developing PD (Hertzman et al., 1990; Liou et al., 1997; Landrigan et al., 2005; McCormack et al., 2005; Tanner et al., 2011). In a meta-analysis of 19 studies, Priyadarshi et al. (2000) reported an association between high pesticide use and increased risk for PD with combined odds ratio of 2.15 among farmers, people living close to farms, and those exposed to farm animals. Additionally, in a review of 38 case-control studies, Brown et al. (2006) showed a robust relationship between long-term pesticide use and increased risk for developing PD.

Epidemiological studies are problematic in that most of the subjects have been exposed to more than one agent, assessment of chronic exposure is based on recall and that most such studies do not identify subpopulations that are at differential risk. Nevertheless on the first count, one pesticide, paraquat, an herbicide, is a major target of study. For example, Liou et al. (1997) showed chronic exposure to paraquat to be associated with increased risk for PD. Moreover, individuals who are exposed to paraquat are at higher risk for developing PD compared to other herbicides and pesticides (Ritz and Yu, 2000; Dhillon et al., 2008; Gatto et al., 2009; Tanner et al., 2009). Numerous case-control studies show a significant association between the extent of exposure to paraquat and the severity of the disease (Kuopio et al., 1999).

Results of both clinical and epidemiological studies, concerning environmental toxicants are inconsistent; not all of the epidemiological studies support the contribution of the same toxicants in PD (Elbaz et al., 2009; Gatto et al., 2009; Firestone et al., 2010). Gatto et al. (2009) reported that the increased risk for PD was associated not specifically to a single pesticide, but rather to a combination of several pesticides including organophosphorous compounds. In a case-control study conducted by Firestone et al. (2010), no association was found between exposure to industrial toxicants and risk for PD.

Some of the inconsistencies may derive from duration of exposure, diagnostic criteria, bias in case-control subject selection, and lack of control for other confounding factors (Berry et al., 2010; Moretto and Colosio, 2012).

Although epidemiological studies are important tools for determining risk, they can be limited by often failing to take into account the role of individual differences reflected in subpopulations. Identifying subpopulations at different genetic-based risk is one way to improve the study design. Identifying individuals carrying such genotypes is challenging, but possible. One example involves polymorphisms cytochrome P450D6 (CYP2D6; Smith et al., 1992; Elbaz et al., 2004). CYP2D6 is involved in the metabolism of several drugs and toxicants, including insecticides and herbicides. One allele, CYP2D6^∗^4 is implicated in relatively slow metabolism of several pesticides, as an autosomal recessive trait. About 5–10% of white populations are homozygous for the allele and for them the enzyme activity is practically undetectable. Elbaz et al. (2004) revealed a twofold increase in risk for PD who were homozygous for the CYP2D6^∗^4 allele (i.e., poor metabolizers) and who were exposed to pesticides. The study population included farmers or people who used pesticides frequently for gardening. Alternatively, normal metabolizers exposed to pesticides showed a slight increase in risk for PD compared to poor metabolizers not exposed to pesticides. These results highlight the importance of gene-environment interactions relevant to neurotoxicology.

Additional evidence to support the importance of host susceptibility is provided by the results reported by Goldman et al. (2012). Glutathione *S*-transferases (GSTM1, GSTT1) are enzymes involved in detoxification of numerous agents in multiple tissues of the human body including, liver, gut, and brain. These enzymes protect different cells of the body against the consequences of oxidative stress induced by multiple bio-reactions and also PD (Landi, 2000; Hayes et al., 2005). Approximately 50 and 20% of Caucasians are homozygous for gene variants, M1 (GSTM1^∗^0) and T1 (GSTT1^∗^0) genotypes, respectively, (Garte et al., 2001). Both variants confer lack of enzyme activity. Goldman et al. (2012) showed that individuals homozygous for GSTT1^∗^0 and exposed to paraquat had an odds ratio for PD risk of 11.1 compared to people with GSTT1 and exposed to paraquat with an OR of 1.5. No additional risk for GSTM1 or GSTM1^∗^0 and exposure to PQ was reported in the study.

### GENOME-WIDE ASSOCIATION STUDIES

Another approach to understanding individual differences in disease and that might have appeal here is the genome-wide association study approach, or GWAS. This approach compares 100s of 1000s or more polymorphic genomic markers in large samples humans with variable phenotypes. This approach has been particularly useful for identifying genetic underpinnings of complex diseases such as restless leg syndrome, (Winkelmann et al., 2007) and familial PD (Soto-Ortolaza et al., 2013). Application of GWAS to toxicology can be illustrated by the work of Pierce et al. (2012). Arsenic contamination of water and soil has been a long-standing problem in Bangladesh and Pierce et al., reported increased signs of differential sensitivity to As poisoning (skin lesions) associated with polymorphisms in arsenite methyltransferase (*As3MT*) one gene known to code for a protein involved in arsenic metabolism. A nearby gene on the same chromosome indicated by the same study may in fact be a gene that regulates the expression of *As3MT.* Whether GWAS is a useful approach to the study of individual differences in response to environmental toxicants remains to be seen. As stated by Zhou and Pearson (2013), GWAS applied to adverse drug reactions (and likely toxicology) may be problematic because of usually small sample sizes and also based on their observation that allelic variants associated with drug responses tend to be quite a bit fewer in number than allelic variants associated with common diseases. The success of the Pierce et al., study is probably attributed to the rather large sample size, the widespread arsenic contamination and the involvement of one or more major genes. Thus, large samples, widespread exposure, well-defined phenotypes, and genes that have major influence on the affected phenotype are important for GWAS studies. This also defines the limitations of GWAS studies in toxicogenetics as many of the effects are polygenic with small additive effects from each of the genes.

While including susceptible subpopulations in epidemiological studies is one way to refine our understanding of individual differences in susceptibility to toxicants, the underlying mechanisms are oftentimes difficult to assess. Complementary to epidemiological studies, animal models can help to elucidate the basis for genetic-based individual differences in susceptibility.

### ANIMAL MODELS IN TOXICOGENETICS – TWO COMPLEMENTARY APPROACHES

Genetic modification is used to create research animals either lacking in function or amplified function in one or more genes. Sometimes, the relevant phenotype is known and sometimes left for discovery. Focusing on the gene initially is often called reverse genetic analysis. Alternatively, genetic analysis can focus on specific, well-defined phenotypes initially and then to a search for relevant genes. This is often termed, “forward genetic analysis.” An elegant description of both may be found in Alonso and Ecker (2006).

### FORWARD GENETIC ANALYSIS OF TOXICITY AS COMPLEX TRAIT

**FIGURE 1:**
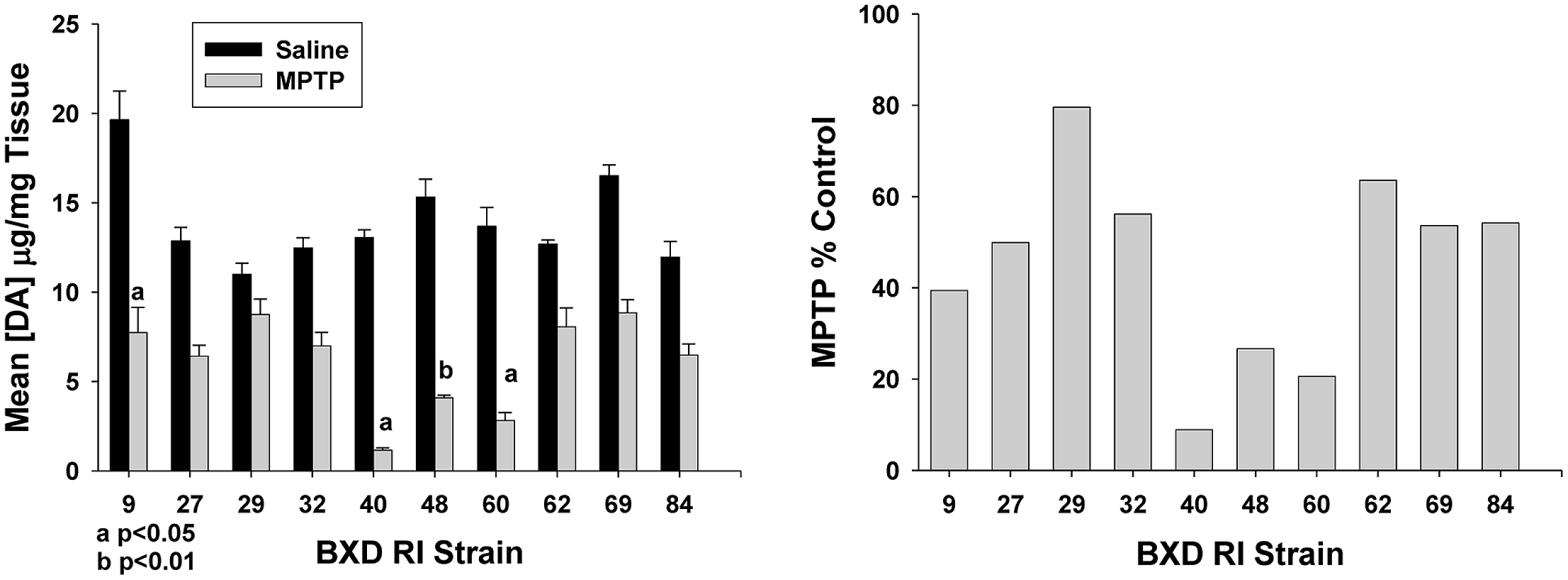
**Effect of 1-methyl-4-phenyl-1,2,3,6-tetrahydropyridine (MPTP) on dopamine (DA) concentration in the caudate-putamen in 10 BXD recombinant inbred mouse strains.** Male mice were injected s.c. with 12.5 mg kg^-1^ of the pro-neurotoxin, MPTP (vs. saline) and sacrificed 48 h later. DA was assayed from tissue homogenates by HPLC. Experimental and control values (upper panel), normalized to tissue weights, are expressed as means ± SEM. The lower panel presents the effect of MPTP expressed as % control. ANOVA revealed a significant main effect of strain, MPTP and a significant interaction (*F*_9,77_ = 11.25; *F*_1,77_ = 445.61; *F*_9,77_ = 9.05, respectively; all *p* < 0.001). Reprinted from Jones et al. (2013).

Findings from epidemiological studies would implicate many if not most effects of environmental toxicants to be complex traits, i.e., effects influenced by several genes and their interaction with the environment. For example, consider our findings from Jones et al. (2013) on the effects of 1-methyl-4-phenyl-1,2,3,6-tetrahydropyridine (MPTP) on striatal DA in BXD recombinant inbred mice (**Figure 1**). These 10 inbred strains (from among more than 100 such strains) were derived from C57BL/6J (B) and DBA/2J (D) parental inbred strains. F_1_ hybrid mice from these two strains were bred *inter se* to produce families that were inbred brother to sister for 20 or more generations in order to recombine and fix alleles. Allelic differences between the two parental strains are now distributed throughout these new strains, the BXD recombinant inbred strains.

The left panel of **Figure 1** illustrates striatal DA concentrations in 10 BXD strains injected with saline (control) or 12.5 mg kg^-1^ MPTP (s.c.) and the right panel illustrates the effect of MPTP on DA loss, expressed as percent of control. As can be seen in the left panel, there is about a 1.5+-fold difference in DA concentration among saline-treated animals between the highest value (BXD 9) and the lowest (BXD 29). In the right panel, we see the extent of DA loss to be highly variable among the strains with BXD 40 being the most sensitive strain and BXD 29 being the least sensitive. MPTP neurotoxicity is achieved through its metabolism to 1-methyl-4-phenylpyridinium MPP+ by astrocytes. MPP+ is then taken up into neurons by the DA transporter where it then causes destruction of those neurons. Knowing that the production of MPP+ is crucial to neurological destruction, we asked the question as to whether the extent of DA loss in the striatum is directly related to the amount of MPP+ produced. Comparing the mean loss of DA with the mean production of MPP+ the correlation was -0.15 and not significant at 7 degrees of freedom (Jones et al., 2014b). This illustrates one of the advantages of forward genetic analysis using a systems perspective.

The continuous variation in DA and DA loss from MPTP treatment shows that MPTP toxicity is polygenic, thus producing individual differences in susceptibility. Had there been only one significant gene, the results would have lined up against each allele from the two progenitor strains – i.e., shown Mendelian trait characteristic. Also, forward genetic analysis allowed us to compare several MPTP-related phenotypes across the same strains so that we could get a view of MPTP toxicity from a systems biology perspective (Jones et al., 2013). Finally, using forward genetic analysis, we can compare our phenotypes against polymorphic markers in the mouse genome and also with gene expression in various tissues. Finally, forward genetic analysis is particularly well-suited for discovery of genes underlying complex phenotypes.

Recombinant inbred mouse strains are particularly useful in forward genetic analysis, and outbred stocks are valuable as well. Two new resources available now are the Collaborative Cross which promises to deliver several hundred recombinant inbred strains derived from eight strains including wild-derived stocks (Threadgill et al., 2011). These strains present a distinct advantage over extant recombinant strains by capturing more of the genetic variation found in mouse populations, compared to those recombinant strains derived from two inbred strains. The goal is to genotype all of these strains and to conduct and publish omnibus gene expression profiles in multiple tissues. Another resource, the Diversity Outbred mouse population is derived from the same eight strains, but not inbred (Churchill et al., 2012). The Collaborative Cross provides a unique platform for systems genetic analysis of complex traits and the Diversity Outbred offers precise mapping of complex traits.

### WHAT ABOUT REVERSE GENETIC ANALYSIS?

The distinction between forward and reverse genetics is somewhat arbitrary, but useful in focusing attention on what to examine first, phenotypes or genes. Gene modification can be particularly useful when working with well-established biochemical pathways and whose genes are known. A recent study by Choi et al. (2010) showed that repression of the gene that produces c-Jun-N-terminal kinase three reduced paraquat- and rotenone- related destruction of DA neurons – an example of specific gene targeting. A recent review by Eastmond et al. (2013) present evidence in the toxicogenetics of cancer that the use of genetically modified organisms may not be an efficient method for the detection of carcinogenic toxicants. We propose that genetically modified organisms may indeed be employed in the verification of candidate genes nominated via forward genetic analysis.

### PUTTING IT ALL TOGETHER

For the most part, we may consider responses to toxicants as complex traits; that is to say, for most individuals, targeted phenotypes are under the influence of multiple genes interacting with each other and with the environment. This means that most toxicant-related, phenotype-relevant genes are many and with small and possibly (hopefully) additive effects. Less commonly, we might expect to identify individuals who show a large effect produced by a rare genetic variant. Polygene identification in the former leads to difficulty in understanding which genes do what relative to the phenotype and in the latter case, sampling may miss those carrying the rare variant altogether. GWAS studies and forward genetics studies in animals can be complementary and informative. For example, Winkelmann et al. (2007) and Stefansson et al. (2007) each reported GWAS studies of individuals with restless legs syndrome and periodic limb movements that identified associated genetic markers near *BTBD9* gene in humans. Restless legs syndrome and periodic limb movements are associated with low iron in the substantia nigra and related DA dysfunction. When we conducted a study of iron concentration in ventral midbrain of mice (Jones et al., 2003), we noticed a weak QTL near *Btbd9* in the mouse genome and remarked on this in a subsequent article (Jones et al., 2008). DeAndrade et al. (2012) were able to produce mice with mutations in *Btbd9* similar to those seen in humans and observed decreased iron, sleep disturbances and abnormal movements similar to human RLS. In this case the mouse researchers were able to capitalize on findings from GWAS, partly confirm through forward genetics and then finally target the gene for manipulation and eventual identification of underlying mechanism.

### GENERAL COMMENTS CONCERNING RODENTS IN TOXICOLOGY

There are a number of valid criticisms about *in vivo* assessment of toxicants. Of utmost importance is whether the studies in animals provide useful information concerning humans. A recent article outlined the advantages and drawbacks of the two-year bioassay (standardized testing as developed more than 40 years ago) of proposed carcinogens in rodents Marone et al. (2013). In fact, the Marone article joins a number of others expressing some dissatisfaction with the assay, with criticisms including time, large numbers of animals, often single-endpoints without concern for the rest of the biological system and finally cost-effectiveness concerning informing human carcinogenesis. These problems lead some to question the value of animal studies in this effort altogether. Among ethical concerns about animal research in general and toxicology specifically, is the effort to refine, reduce and to replace (the three Rs). We propose that the use of genetically diverse animals (genetic reference populations of rats and mice) for initial screening for differences in response to toxicants can identify genes and biochemical pathways underlying the differences. Follow-up genetic manipulation studies can offer proof-of concept. This approach has the potential to refine methods and therefore reduce animal numbers. Moreover, a systems study of toxicity in rodents can further elucidate the impact of toxicant exposure. In our recent MPTP study (Jones et al., 2013), we performed principal components analysis on a number of DA-related measures and then were able to relate the composite index to a network of co-expressed genes and possible involved biochemical pathways.

## CONCLUSION

Now that people are living longer, numerous chronic diseases that would be considered to be rare in earlier times are becoming more common. The remarkable increase in life expectancy over the past 100 years accompanied by longer exposures to environmental toxicants underscore the importance of toxicological research. Better identification of host characteristics in epidemiological and GWAS studies that affect toxicity to specific agents, coupled with carefully planned experiments in genetic reference populations in animals (Ermann and Glimcher, 2012) can lead to better prediction of individuals at risk and may even facilitate better prevention and treatment post exposure.

## Conflict of Interest Statement

The authors declare that the research was conducted in the absence of any commercial or financial relationships that could be construed as a potential conflict of interest.

## References

[B1] Alonso J. M. Ecker J. R. (2006). Moving forward in reverse: genetic technologies to enable genome-wide phenotypoic screens in *Arabidopsis*. *Nat. Rev. Genet.* 7 524–536 10.1038/nrg1893 16755288

[B2] Berry C. Vecchia C. L. Nicotera P. (2010). Paraquat and Parkinson’s disease. *Cell Death Differ.* 17 1115–1125 10.1038/cdd.2009.217 20094060

[B3] Brown T. P. Ramsby P. C. Capleton A. C. Rushton L. Levy L. S. (2006). Pesticides and Parkinson’s disease- is there a link? *Environ. Health* 114 165–164 10.1289/ehp.8095 PMC1367825 16451848

[B4] Choi W. S. Abel G. Klintworth H. Flavell R. A. Xia Z. (2010). JNK3 mediates paraquat- and rotenone-induced dopaminergic neuron death. *J. Neuropathol. Exp. Neurol.* 69 511–520 10.1097/NEN.0b013e3181db8100 20418776 PMC3061491

[B5] Churchill G. A. Gatti D. M. Munger S. C. Svenson K. L. (2012). The diversity outbred mouse population. *Mamm. Genome* 23 713–718 10.1007/s00335-012-9414-2 22892839 PMC3524832

[B6] DeAndrade M. P. Johnson R. L. Jr. Unger E. L. Zhang L. van Groen T. Gamble K. L. (2012). Motor restlessness, sleep disturbances, thermal sensory alterations and elevated serum iron levels in Btbd9 mutant mice. *Hum. Mol. Genet.* 21 3984–3992 10.1093/hmg/dds221 22678064 PMC3428151

[B7] Dhillon A. S. Tarbutton G. L. Levin J. L. Plotkin G. M. Lowry L. K. Nalbone J. T. (2008). Pesticide/ environmental exposures and Parkinson’s disease in east Texas. *J. Agromedicine* 13 37–48 10.1080/10599240801986215 19042691

[B8] Eastmond D. A. Vulimiri S. V. French J. E. Sonawane B. (2013). The use of genetically modified mice in cancer risk assessment: challenges and limitations. *Crit. Rev. Toxicol.* 43 611–631 10.3109/10408444.2013.822844 23985072 PMC4457504

[B9] Elbaz A. Clavel J. Rathouz P. J. Moisan F. Galanaud J. P. Delemotte B. (2009). Professional exposure to pesticides and Parkinson disease. *Ann. Neurol.* 66 494–504 10.1002/ana.21717 19847896

[B10] Elbaz A. Levecque C. Clavel J. Vidal J. S. Richard F. Amouyel P. (2004). CYP2D6 polymorphism, pesticide exposure, and Parkinson’s disease. *Ann. Neurol.* 55 430–434 10.1002/ana.20051 14991823

[B11] Ermann J. Glimcher L. H. (2012). After GWAS: mice to the rescue? *Curr. Opin. Immunol.* 24 564–570 10.1016/j.coi.2012.09.005 23031443 PMC3631559

[B12] Firestone J. A. Lundin J. I. Powers K. M. Smith-Weller T. Franklin G. M. Swanson P. D. (2010). Occupational factors and risk of Parkinson’s disease: a population-based case-control study. *Am. J. Ind. Med.* 53 217–223 10.1002/ajim.20788 20025075 PMC3299410

[B13] Garte S. Gaspari L. Alexandrie A. K. Ambrosone C. Autrup H. Autrup J. L. (2001). Metabolic gene polymorphism frequencies in control populations. *Cancer Epidemiol. Biomarkers Preve.* 10 1239–1248 11751440

[B14] Gatto N. M. Cockburn M. Bronstein J. Manthripragada A. D. Ritz B. (2009). Well-water consumption and Parkinson’s disease in rural California. *Environ. Health Perspect.* 117 1912–1918 10.1289/ehp.0900852 20049211 PMC2799466

[B15] Goldman S. M. Kamel F. Bhudhikanok G. S. Ross G. W. Hoppin J. A. Korell M. (2012). Genetic modification of the association of paraquat and Parkinson’s disease. *Mov. Disord.* 27 1652–1658 10.1002/mds.25216 23045187 PMC3572192

[B16] Hayes J. D. Flanagan J. U. Jowsey I. R. (2005). Glutathione transferases. *Annu. Rev. Pharmacol. Toxicol.* 45 51–88 10.1146/annurev.pharmtox.45.120403.095857 15822171

[B17] Hertzman C. Wiens M. Bowering D. Snow B. Calne D. (1990). Parkinson’s disease: a case-control study of occupational and environmental risk factors. *Am. J. Ind. Med.* 17 349–355 10.1002/ajim.4700170307 2305814

[B18] Jones B. C. Huang X. Mailman R. B. Lu L. Williams R. W. (2014a). The perplexing paradox of paraquat: the case for host-based susceptibility and postulated neurodegenerative effects. *J. Biochem. Mol. Toxicol.* 28 191–197 10.1002/jbt.21552 24599642 PMC4677573

[B19] Jones B. C. O’Callaghan J. P. Lu L. Williams R. W. Alam G. Miller D. B. (2014b). Genetic correlational analysis reveals no association between MPP+ and the severity of striatal dopaminergic damage following MPTP treatment. *Neurotoxicol. Teratol.* 10.1016/j.ntt.2014.08.005 [Epub ahead of print] PMC7401901 25192776

[B20] Jones B. C. Miller D. M. O’Callaghan J. P. Lu L. Unger E. L. Alam G. (2013). Systems analysis of genetic variation in MPTP neurotoxicity in mice. *J. Neurotox.* 37 26–34 10.1016/j.neuro.2013.03.010 PMC4615717 23558233

[B21] Jones B. C. Reed C. L. Hitzemann R. Wiesinger J. A. McCarthy K. A. Buwen J. P. (2003). Quantitative genetic analysis of ventral midbrain and liver iron in BXD recombinant inbred mice. *Nutr. Neurosci.* 6 369–377 10.1080/10284150310001624192 14744041

[B22] Jones L. C. Earley C. J. Allen R. P. Jones B. C. (2008). Of mice and men, periodic limb movements and iron: how the human genome informs the mouse genome. *Genes Brain Behav.* 7 513–514 10.1111/j.1601-183X.2008.00400.x 18363860

[B23] Kuopio A. M. Marttila R. J. Helenius H. Rinne U. K. (1999). Environmental risk factors in Parkinson’s disease. *Mov. Diord.* 14 928–939 10.1002/1531-8257(199911)14:6<928::AID-MDS1004>3.0.CO;2-Z 10584666

[B24] Landi S. (2000). Mammalian class theta GST and differential susceptibility to carcinogens: a review. *Mutat. Res.* 463 247–283 10.1016/S1383-5742(00)00050-8 11018744

[B25] Landrigan P. J. Sonawane B. Butler R. N. Trasande L. Callan R. Droller D. (2005). Early environmental origins of neurodegenerative disease in later life. *Environ. Health Perspect.* 113 1230–1233 10.1289/ehp.7571 16140633 PMC1280407

[B26] Liou H. H. Tsai M. C. Chen C. J. Jeng J. S. Chang Y. C. Chen S. Y. (1997). Environmental risk factors and Parkinson’s disease. *Neurology* 48 1583–1588 10.1212/WNL.48.6.1583 9191770

[B27] Marone P. A. Hall W. C. Hayes A. W. (2013). Reassessing the two-year rodent carcinogenicity bioassay: a review of the applicability to human risk and current perspectives. *Regul. Toxicol. Pharmacol.* 68 108–118 10.1016/j.yrtph.2013.11.011 24287155

[B28] McCormack A. L. Atienza J. G. Johnston L. C. Anderaen J. K. Vu S. Di Monte D. A. (2005). Role of oxidative stress in paraquat-induced dopaminergic cell degeneration. *J. Neurochem.* 93 1030–1037 10.1111/j.1471-4159.2005.03088.x 15857406

[B29] Moretto A. Colosio C. (2012). The role of pesticide exposure in the genesis of Parkinson’s disease: epidemiological studies and experimental data. *Toxicology* 307 24–34 10.1016/j.tox.2012.11.021 23246862

[B30] Pierce B. Kibriya M. Tong L. Jasmine F. Argos M. Roy S. (2012). Genome-wide association study identifies chromosome 10q24.32 variants associated with arsenic metabolism and toxicity phenotypes in bangladesh *PLoS Genet.* 8:e1002522 10.1371/journal.pgen.100252 PMC3285587 22383894

[B31] Priyadarshi A. Khuder S. A. Schaub E. A. Shrivastava S. (2000). A meta-analysis of Parkinson’s disease and exposure to pesticides. *Neurotoxicology* 21 435–440 11022853

[B32] Ritz B. Yu F. (2000). Parkinson’s disease mortality and pesticide exposure in California 1984-1994. *Int. J. Epidemiol.* 29 323–329 10.1093/ije/29.2.323 10817132

[B33] Smith C. A. D. Wolf C. R. Gough A. C. Spurr N. K. Leigh P. N. Summers S. B. (1992). Debrisoquine hydroxylase gene polymorphism and susceptibility to Parkinson’s disease. *Lancet* 1 1375–1377 10.1016/0140-6736(92)91196-F 1350805

[B34] Soto-Ortolaza A. I. Heckman M. G. Labbé C. Serie D. J. Puschmann A. Rayaprolu S. (2013). GWAS risk factors in Parkinson’s disease: LRRK2 coding variation and genetic interaction with PARK16. *Am. J. Neurodegener. Dis.* 2 287–299 24319646 PMC3852568

[B35] Stefansson H. Rye D. B. Hicks A. Petursson H. Ingason A. Thorgeirsson T. E. (2007). A genetic risk factor for periodic limb movements in sleep. *N. Eng. J. Med.* 357 639–647 10.1056/NEJMoa072743 17634447

[B36] Tanner C. M. Kamel F. Ross G. W. Hoppin J. A. Goldman S. M. Korell M. (2011). Rotenone, paraquat, and Parkinson’s disease. *Environ. Health Perspect.* 119 866–872 10.1289/ehp.1002839 21269927 PMC3114824

[B37] Tanner C. M. Ross G. W. Jewell S. A. Hauser R. A. Jankovic J. Factor S. A. (2009). Occupation and risk of parkinsonism: a multicenter case-control study. *Arch. Neurol.* 66 1106–1113 10.1001/archneurol.2009.195 19752299

[B38] Threadgill D. W. Miller D. R. Churchill G. A. de Villena F. P. (2011). The collaborative cross: a recombinant inbred mouse population for the systems genetic era. *ILAR J.* 52 24–31 10.1093/ilar.52.1.24 21411855

[B39] Winkelmann J. Schormair B. Lichtner P. Ripke S. Xiong L. Jalilzadeh S. (2007). Genome-wide association study of restless legs syndrome identifies common variants in three genomic regions. *Nat. Genet.* 39 1000–1006 10.1038/ng2099 17637780

[B40] Zhou K. Pearson E. R. (2013). Insights from genome-wide association studies of drug response. *Annu. Rev. Pharmacol. Toxicol.* 53 299–310 10.1146/annurev-pharmtox-011112-140237 23072379

